# Eculizumab's Unintentional Mayhem: A Systematic Review

**DOI:** 10.7759/cureus.25640

**Published:** 2022-06-03

**Authors:** Ravneet K Dhanoa, Ramaneshwar Selvaraj, Shoukrie I Shoukrie, Anam Zahra, Jyothirmai Malla, Tharun Yadhav Selvamani, Sathish Venugopal, Ranim K Hamouda, Pousette Hamid

**Affiliations:** 1 Internal Medicine/Hematology, California Institute of Behavioral Neurosciences & Psychology, Fairfield, USA; 2 Internal Medicine/Family Medicine/General Surgery, California Institute of Behavioral Neurosciences & Psychology, Fairfield, USA; 3 Orthopaedics/Traumatology, California Institute of Behavioral Neurosciences & Psychology, Fairfield, USA; 4 Surgery, California Institute of Behavioral Neurosciences & Psychology, Fairfield, USA; 5 Internal Medicine, California Institute of Behavioral Neurosciences & Psychology, Fairfield, USA; 6 General Surgery, California Institute of Behavioral Neurosciences & Psychology, Fairfield, USA; 7 Neurology, California Institute of Behavioral Neurosciences & Psychology, Fairfield, USA

**Keywords:** atypical hemolytic uremic syndrome, piga gene defect, paroxysmal nocturnal hemoglobinuria, neisseria meningitidis, meningococcemia, complement inhibitor, eculizumab

## Abstract

Eculizumab, first-line therapy for paroxysmal nocturnal hemoglobinuria (PNH) and atypical hemolytic uremic syndrome (aHUS), has infectious side effects in addition to its therapeutic benefits. This study aims to discuss the mechanism of development of infections, prevention, and timely treatment to prevent complications such as septic shock and mortality. The study was conducted using the Preferred Reporting Items for Systematic Reviews and Meta-analyses (PRISMA) checklist and reporting guidelines for systematic review. Inclusion and exclusion criteria were determined. A total of 10 research papers were extracted after exploring Pubmed and Google Scholar from 2001 to 2021. The New Castle Ottawa Questionnaire for non-randomized clinical trials and the National Institutes of Health (NIH) quality assessment tool for case reports and case series were used to assess the risk of bias. The studies included in this systematic review describe infections with *Neisseria meningitidis*, *Neisseria* *gonorrhoeae*, unusual *Neisseria *species, *Moraxella lacunata*, and *Pseudomonas aeruginosa*. The main goal of this review is to impress upon the seriousness of the infectious complications associated with eculizumab. Health care providers should maintain a high index of suspicion for early identification and treatment.

## Introduction and background

"But however secure and well-regulated civilized life may become, bacteria, protozoa, viruses, infected fleas, lice, ticks, mosquitoes, and bed bugs will always lurk in the shadows ready to pounce when neglect, poverty, famine, or war lets down the defenses" - Hans Zinsser [1].

Eculizumab, a monoclonal antibody, approved by the United States Food and Drug Administration (FDA) for three indications: paroxysmal nocturnal hemoglobinuria (PNH), atypical hemolytic uremic syndrome (aHUS), and anti-acetylcholine receptor-positive generalized myasthenia gravis (gMG), is a complement inhibitor that inhibits the complement C5 that normally mounts a defense response against the encapsulated bacteria, *Neisseria meningitidis (N. meningitidis)*, causing the bacteria to take advantage of this vulnerable situation and lead to meningitis, septic shock, and severe end-organ damage [2].

PNH is a rare hematologic disorder with an incidence of 15.9 individuals per million worldwide with most of the patients falling in the range of 30-40 years. It is an X-linked disease with a mutation in the phosphatidylinositol glycan class A (*PIGA*) gene producing a deficiency in glycosylphosphatidylinositol (GPI) protein, which anchors protein moieties such as CD55 and CD59 that further block the activity of membrane attack complex (C5b-C9). Due to loss of complement inhibition, RBCs become vulnerable to destruction by membrane attack complex resulting in hemolytic anemia. Thus, PNH presents with hemolytic anemia, hemoglobinuria, thrombosis, renal insufficiency, and aplastic anemia [3].

aHUS is a disorder of dysregulation of the alternative complement pathway in which there is uncontrolled activation of the alternative complement pathway resulting in inflammation, which produces endothelial injury, platelet activation and aggregation, leukocyte recruitment, and pro-coagulative signaling leading to thrombosis. Hence, it is called thrombotic microangiopathy, which is characterized by thrombocytopenia, hemolytic anemia, acute kidney injury, and other end-organ damage. Without treatment, it may progress to renal failure with mortality occurring in up to 7% of patients after one year [4].

Eculizumab (Soliris®, Alexion Pharmaceuticals, Boston, Massachusetts, United States), a complement component 5 targeted agent is a recognized therapeutic modality for PNH [5] and is the only treatment agent approved by the FDA and European Medicines Agency as well as 49 countries worldwide for treating aHUS [4]. Though eculizumab is very beneficial for the aforementioned diseases, it is associated with some major adverse effects. The prescribing information for the FDA-approved eculizumab includes a black box warning of the elevated risk of *N. meningitidis* infection. Since membrane attack complex is required for the serum meningococcal bactericidal activity, its inhibition by eculizumab poses a risk of a full-fledged infection with meningococcus [6]. The risk for invasive meningococcal infection increases by 1000-2000 times [5]. The symptoms caused by *N. meningitidis* can range from fever, vomiting, headache, cold hands and feet, pallor or mottling of the skin to hemorrhagic rash, impaired consciousness, and death. The case fatality rate of meningococcemia is 40%. The incidence of *N. meningitidis* infection varies between 0.11 and 2.8 per 100,000 person-years in Europe, Australia, New Zealand, South America, and North America and 112 per 100,000 person-years in the African meningococcal belt zone [7]. Meningococcal vaccination should be provided two weeks before the initiation of eculizumab to prevent infection with meningococcus [8]. 

Based on the case reports of disseminated gonococcal infections (DGI) in patients treated with eculizumab, we need to study and find out the association between *Neisseria gonorrhoeae (N. gonorrhoeae) *infections and the use of eculizumab [5,9]. Not only *N. gonorrhoeae*, but there have also been cases where invasive infections have been caused by non-meningococcal, non-gonococcal *Neisseria* species like *N. mucosa, N. subflava*, and *N. cinerea* in association with eculizumab. These organisms are commensal and can lead to endocarditis, meningitis, pneumonia, peritonitis, septic arthritis, etc [2]. Another commensal organism that has been a culprit in causing infection with the use of eculizumab is *Moraxella lacunata.* It is a gram-negative coccobacillus found in the upper respiratory tract and conjunctiva and is known to cause bacteremia and systemic inflammatory response syndrome (SIRS) in one of the cases receiving eculizumab for aHUS [10]. There have been instances where infections by *Pseudomonas aeruginosa*, *Aspergillus niger*, polyomavirus John Cunningham (JC) virus, and herpes simplex virus have been documented while the patient was treated with eculizumab [11]. Although the mechanism is not clear, there is a dire need to consider the risk of infections other than *N. meningitidis* in association with the use of eculizumab.

This systematic review is an up-to-date synthesis of various infections that occurred in different countries during different years in patients with PNH/aHUS being treated with eculizumab. Our study builds on previous results by including the analysis of study quality. There is extensive literature related to the adverse effects associated with the use of eculizumab and this information must be summarized to get an overview of the literature. Given the above, the purpose of the current study was to review the qualitative literature and answer two primary research questions: (1) What are the adverse effects associated with the use of eculizumab in PNH/aHUS patients? and (2) Is there a way to prevent/treat these adverse effects?

## Review

Protocol

We conducted our systematic review using Preferred Reporting Items for Systematic Reviews and Meta-Analysis (PRISMA) guidelines [12]. 

Database

We started our research on December 20, 2021, using online libraries as our database. A systematic search of major electronic databases (PubMed and Google Scholar) was conducted by the primary author.

Search strategy

We included the studies describing various infections associated with the use of eculizumab in PNH/aHUS patients. To include pertinent papers, snowballing technique was used. Also, we followed the authors who cited the articles pertinent to our topic. It was conducted independently by two reviewers (R.D. and R.S.) and in the event of disagreement, a third opinion was sought (S.S). Our keywords, medical subject heading (MeSH) search strategies, and each search result have been described in Table 1.

**Table 1:** Database search results using regular keywords and MeSH strategy MeSH: Medical subject heading search strategy

Keywords	Database	Initial Search Results
Eculizumab OR Complement inhibitor AND Meningococcemia OR Neisseria Meningitidis AND Paroxysmal Nocturnal Hemoglobinuria OR PIGA gene defect AND Atypical Hemolytic Uremic Syndrome AND (( "Complement Inactivating Agents/adverse effects"[Majr] OR "Complement Inactivating Agents/immunology"[Majr] AND (( "Neisseria meningitidis/drug effects"[Majr] OR "Neisseria meningitidis/genetics"[Majr] AND (( "Hemoglobinuria, Paroxysmal/blood"[Majr] OR "Hemoglobinuria, Paroxysmal/drug therapy"[Majr]	Pubmed	908
	Google Scholar	2380

Inclusion criteria

We selected free full articles in the English language. Only human studies that were done in the last 20 years from January 1, 2001, to December 31, 2021, were included. The population included has PNH or aHUS, with eculizumab being their only treatment modality.

Exclusion criteria

We excluded any population not having PNH/aHUS and any population having PNH/aHUS being treated with a complement inhibitor other than eculizumab. We excluded animal studies, articles published in languages other than English, and studies done before the last 20 years. Letters and Editorials were excluded.

Quality assessment tools

We used the New Castle Ottawa questionnaire for non-randomized clinical trials and the National Institutes of Health (NIH) quality assessment tool for case reports and case series. Studies of low quality were excluded. This was conducted independently by two reviewers (R.D. and R.S.) and in the event of disagreement, a third opinion was sought (S.S).

Data collection

Data were extracted from the final articles by two independent reviewers (R.D. and R.S). Any discrepancies between the reviewers were resolved through discussion with a third author (S.S).

Results

**Figure 1:**
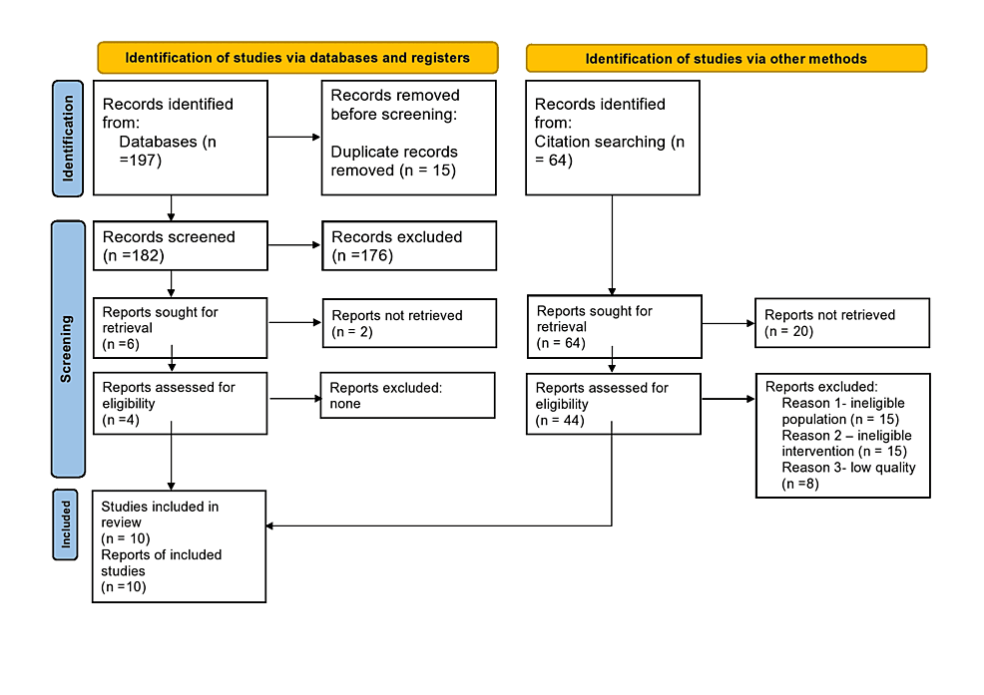
PRISMA flow diagram outlining the screening and selection process of articles obtained from different databases PRISMA: Preferred Reporting Items for Systematic Reviews and Meta-Analyses

In total, 261 articles were identified; 197 from databases (157 from PubMed, 40 from Google Scholar), and 64 via citation searching. After duplicates were removed, 182 records were screened (abstract and title) and four records were assessed for eligibility. Of the 64 articles found via citation searching, 44 records were assessed for eligibility. After further assessment, it was determined that the review inclusion criteria were met by 10 studies [2,5,6,8,9,10,11,12,13,14]. The PRISMA flow diagram is shown in Figure 1.

The characteristics of the included 10 articles are shown in Table 2.

**Table 2:** The characteristics of included studies BMC: BioMed Central

Author	Journal	Publication Year	Type of Infection	Type of Research
Saito et al. [5]	International Journal of General Medicine	2020	Disseminated gonococcal infection	Case Report and Literature Review
Ladhani et al. [13]	BMC Infectious diseases	2019	Invasive meningococcal disease	Case Series
Walsh et al. [14]	Infection	2018	*Neisseria cinerea* bacteremia	Case Report
Nolfi-Donegan et al. [6]	Emerging Infectious Diseases	2018	Neisseria meningitidis	Case Report
Langereis et al. [15]	Blood Advances	2020	Neisseria meningitidis	Clinical Trial
Kawakami et al. [11]	Internal Medicine	2018	Pseudomonas aeruginosa	Case Report
Hublikar et al. [8]	Sexually Transmitted Diseases	2014	Disseminated gonococcal infection	Case Report
Crew et al. [9]	Clinical Infectious Diseases	2019	Disseminated gonococcal infection	Case Series
Crew et al. [2]	Journal of Infection	2019	Unusual *Neisseria* species	Case Series
Bicoll et al. [10]	Clinical Medicine Insights: Pediatrics	2021	*Moraxella lacunata *bacteremia	Case Report

Discussion

This review article will discuss the types of bacterial infections that develop secondary to eculizumab therapy. This research report goes into detail about the clinical presentation of the infections, their prevention, and treatment.

Neisseria meningitidis

The terminal complement pathway inhibition caused by eculizumab increases the risk of invasive meningococcal disease (IMD) [13]. In a cohort study undertaken by Ladhani et al., IMD was found in six patients with PNH and three people with aHUS who were treated with eculizumab. The probability of IMD due to capsular group Y is 1/9, or 11%. Four of the nine IMD events in patients treated with eculizumab were NG or Group E. These strains aren't as virulent as others and are only linked to the carriage [13]. Also, Nolfi-Donegan describes a case report of a patient who developed fatal *N. meningitidis* disease while receiving eculizumab [6]. The characteristics of patients developing IMD are described in Table 3. 

**Table 3:** Manifestation of Neisseria meningitidis infection in patients PNH: Paroxysmal Nocturnal Hemoglobinuria; aHUS: Atypical Hemolytic Uremic Syndrome

Date of infection	Age (years)	Capsular group	Vaccination status	Underlying condition	Treatment
October 2008 [13]	20	B	Immunized	PNH	Penicillin
September 2011 [13]	20	B	Immunized	PNH	Ciprofloxacin
January 2017 [13]	25	Y	Immunized	PNH	Penicillin
October 2009 [13]	40	B	Not known	PNH	None
March 2017 [13]	23	W	Immunized	PNH	Penicillin
September 2015 [13]	22	NG	Immunized	aHUS	Penicillin
May 2016 [13]	20	E	Immunized	aHUS	Not known
December 2014 [13]	20	NG	Immunized	aHUS	Not known
March 2017 [13]	22	NG	Not known	Not known	Not known
March 2016 [6]	16	NG	Immunized	PNH	Ketorolac, Prochlorperazine, Diphenhydramine.

Immunization

Meningococcal ACWY (Men ACWY), a polysaccharide conjugate vaccine against groups A, C, W, and Y, Meningococcal B vaccine (4CMenB) against IMD in infants and toddlers, and two novel broad-spectrum protein-based meningococcal vaccines that have been approved in Europe are available [13]. 

Vaccine failure has been documented after 4CMenB and MenACWY. Patients with complement insufficiency still acquire IMD despite having high post-immunization antibody titers [13,15]. Langereis et al. conducted a clinical experiment employing whole blood killing assay to investigate opsonophagocytic *Neisseria meningitidis* serogroup B (MenB) killing in PNH patients receiving eculizumab who were vaccinated with 4CMenB. Despite IgG and C3 opsonization of the bacterial surface, the whole blood lysis of MenB was not detected after vaccination with the 4CMenB vaccine indicating the need to use antibiotics prophylactically to prevent MenB infections [15]. 

Prophylactic Antibiotics

Prophylactic antibiotics are recommended until two weeks after vaccination, according to the US FDA. The European Society of Clinical Microbiology and Infectious Diseases recommends the use of antibiotics for four weeks after immunization [15]. Penicillin or erythromycin are used in the majority of chemoprophylactic regimens for eculizumab-treated patients. Meningococcal disease caused by strains with penicillin resistance or intermediate sensitivity has been reported despite penicillin chemoprophylaxis [6].

Neisseria gonorrhoeae

FDA did a worldwide search to find reports of *N. gonorrhoeae* infection in individuals receiving eculizumab. The FDA's MedWatch program analyzed pre-and post-marketing safety reports that were stored in either the FDA adverse event reporting system (FAERS) or as part of a safety submission under an investigational new drug (IND) application. Crew et al. used the aforesaid source to establish a case study of nine patients who had *N. gonorrhoeae *infection after using eculizumab as well as Saito et al. and Hublikar et al., who wrote a case report on individuals who developed gonococcal infection with the use of eculizumab [5,8,9]. The features of patients who developed the gonococcal infection are shown in Table 4.

**Table 4:** The characteristics of gonococcal infection in different patients PNH: Paroxysmal Nocturnal Haemoglobinuria; NAAT: Nucleic Acid Amplification Test; aHUS: Atypical Haemolytic Uremic Syndrome; DGI: Disseminated Gonococcal Infection; ID: Infectious Disease; RUQ: Right Upper Quadrant; ICU: Intensive Care Unit; BP: Blood Pressure; PCR: Polymerase Chain Reaction; DNA: Deoxyribonucleic acid; WBC: White blood cell; LDH: Lactate Dehydrogenase; CRP: C-reactive protein; F: Female; M: Male

Age, Sex	Underlying condition	Source of Isolate	Presentation	Treatment	Outcome
22, F [9]	PNH	Positive blood cultures	Not reported	Not reported	Not reported
28, F [9]	PNH	Positive urogenital NAAT, positive blood cultures	Swollen left index finger, fever 103ﹾF, headache	Vancomycin, ceftriaxone followed by ceftriaxone and azithromycin	Resolved
23, F [9]	aHUS	Positive blood cultures; negative NAAT for cervical swab and urine sample; negative culture for rectal swab and throat swab.	One day of fever and rigors	Piperacillin-tazobactam, vancomycin followed by ceftriaxone and azithromycin	Resolved
18, F [9]	aHUS	Not reported	Possible pregnancy during infection	Not reported	Resolved
19, F [9]	PNH	Positive cervical culture	Following cervical culture, the patient developed joint pain, DGI diagnosed via ID consult	Not reported	Diagnosis of endocarditis, thrombotic spleen, intracranial hemorrhage, and thrombosis made followed by death
19, F [9]	PNH	Positive blood cultures, negative DNA amplification tests/cultures from rectal and genital swabs.	Fver, vomiting, RUQ abdominal pain, lightheadedness	Vancomycin, ceftriaxone followed by meropenem.	Respiratory failure which required mechanical ventilation and ICU care: eventually resolved
42, M [9]	PNH	Positive blood cultures	Fever, skin eruption on ankles, knees, wrists, abdomen.	Ciprofloxacin	Resolved
44, F [9]	PNH	Positive blood cultures	Trauma to right middle finger three days before admission, presented with fever, body aches, tiredness, low BP, tubal ovarian abscess ruled out via pelvic ultrasound	Cefepime, levofloxacin, vancomycin, ceftriaxone	ICU care, eventually resolved
28, F [9]	aHUS	Positive blood culture, negative skin biopsy for meningococcus and varicella, PCR of endocervix negative for Gonorrhea	Fever, headache, vomiting, chills, maculopapular rash with macular lesions on arms and thighs	Ceftriaxone	Resolved
28, F [8]	PNH	Positive blood cultures, positive NAAT	Fever, left index finger swollen and warm, soft tissue swelling diagnosed on plain radiograph of left hand	Intravenous vancomycin and ceftriaxone	Resolved
22, M [5]	PNH	Positive venous blood cultures	Fever, headache, mild nausea, dysuria. Labs: Hb- 11.5g/dl ( decreased to 8.3 g/dl after three days, LDH-334, CRP-2.24. Urinalysis – WBC of 10-19/high power field	Ceftriaxone	Resolved

Mechanism of Development of Infection

Gonococci has 70% DNA similarity to *N. meningitidis*. Complement insufficiency has long been reported to be a risk factor for gonococcal infections as it is for *N. meningitidis*. Since eculizumab is a complement inhibitor, it has the potential to cause gonococcal infection [5]. 

Treatment

Uncomplicated gonococcal infections of the cervix, urethra, throat, and rectum should be treated with the CDC-recommended regimen of 250mg ceftriaxone intramuscularly and 1g azithromycin orally in a single dosage. For patients with disseminated gonococcal infection (DGI) presenting as arthritis or arthritis-dermatitis syndrome, 1g intramuscular or intravenous ceftriaxone daily and a single oral dose of 1g azithromycin is recommended. DGI presenting as gonococcal meningitis or endocarditis should be treated with 1-2g intravenous ceftriaxone every 12-24 hours and a one-time dosage of 1g oral azithromycin. For meningitis, parenteral treatment should be continued for 10-14 days; for endocarditis, parenteral medication should be given for four weeks [9].

Prevention

Patients should be informed about the significant risk of DGI. Sexual histories should be obtained, including the number and gender of sex partners as well as the type of sexual contact, as this information will aid in the development of suitable screening recommendations. Safe sex habits such as the regular use of condoms should be encouraged [9].

Unusual Neisseria species

Meningococcus expresses factor H binding protein (fHbp), which attracts the inhibitory complement-regulatory protein factor H (CFH) to the microbial surface, preventing opsonophagocytosis and bacteriolysis. Recently, it was discovered that *N. cinerea* can also express fHBP, which binds CFH with the same affinity as meningococcal fHBP. This organism's immune escape strategy, together with eculizumab-induced suppression of opsonophagocytosis and cell lysis, gives scientific plausibility to how a nonpathogenic organism such as unusual *Neisseria* species can become invasive [14]. Crew et al. searched through the FAERS database and medical literature for reports of non-meningococcal and non-gonococcal *Neisseria* species infection in eculizumab patients. They were able to create a case series consisting of six cases of unusual *Neisseria* species infection [2]. Walsh et al. have also described a case report of a patient with *N. cinerea* infection [14]. The features of patients who became infected with uncommon *Neisseria* species are listed in Table 5.

**Table 5:** The characteristics of infection with unusual Neisseria species PNH: Paroxysmal Nocturnal Hemoglobinuria; aHUS: Atypical Hemolytic Uremic Syndrome; BP: Blood Pressure; Hb: Hemoglobin; ED: Emergency Department; ER: Emergency Room;

Age, Sex	Neisseria Species	Underlying condition	Presentation	Source of isolate	Treatment	Outcome
4, M [2]	N. flavescens (subflava)	aHUS	Febrile neutropenia with sepsis	Data not reported	Antibiotics used, but not specified	Infection resolved
17, F [2]	N. cinerea	PNH	Temp-39.5ﹾC, BP-70/40, septic shock, abdominal pain, vomiting, Hb-6.5g/dl, elevated creatinine	N. cinerea growth spotted in blood culture. Gall bladder thickening noticed on Abdominal “eco scan”	Ceftriaxone and metronidazole	Infection resolved
6, M [2]	N. sicca (mucosa)/ subflava	aHUS	Fever 100.9ﹾ(no units), cough at presentation in clinic. Patient sent to ED, condition reported as sepsis	N. sicca/subflava growth noticed on blood cultures from subcutaneous port	Ceftriaxone in clinic, vancomycin, and ceftriaxone in ER followed by ceftriaxone for seven days	Infection resolved
38, F [2]	N. cinerea	aHUS	The physicians ruled out any presence of bacterial infection except for the presence of warmth, mild tenderness to palpation at arteriovenous fistula site in left upper extremity. Hospitalized due to positive blood cultures	N. cinerea growth on three sets of peripheral blood cultures	Cefepime x 14 days	Infection resolved
13, M [2]	N. sicca (mucosa/ Subflava)	PNH	Eculizumab x 12 weeks before bone marrow transplant followed by febrile neutropenia for seven days.	N. sicca/subflava positive on “central line culture”	Piperacillin/tazobactam	Infection resolved
38, F [2]	N. sicca (mucosa)	Catastrophic antiphospholipid syndrome - compassionate use	Diagnosed as “bacterial peritonitis“	Peritoneal fluid culture	Antibiotics used, but not specified	Infection resolved
38, F [14]	N. cinerea	Postpartum aHUS	Bacteremia	Blood cultures positive for N. cinerea	Cefepime x 14 days followed by penicillin prophylaxis	Infection resolved

Pseudomonas aeruginosa

**Figure 2:**
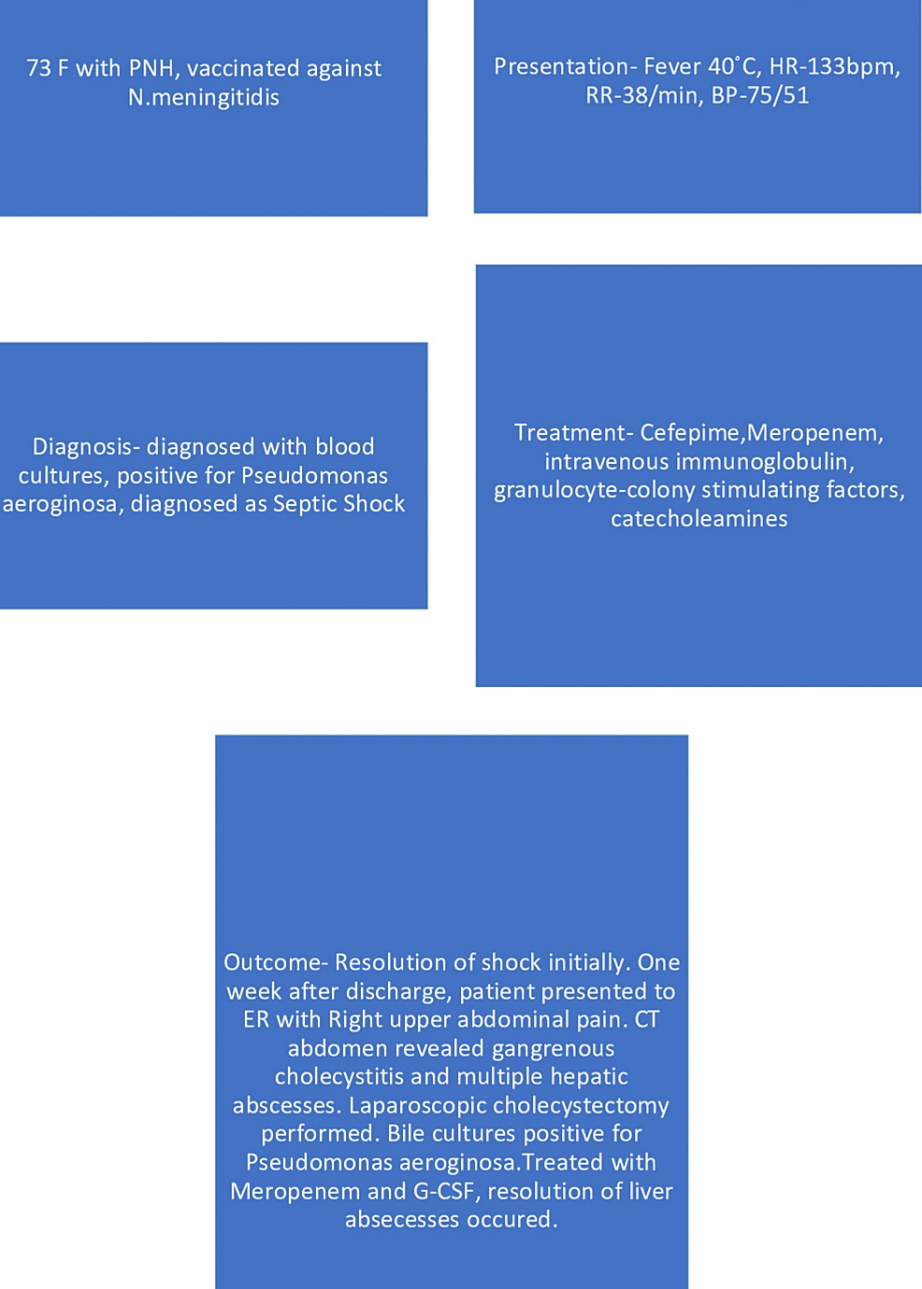
Description of Pseudomonas aeroginosa infection PNH: Paroxysmal Nocturnal Hemoglobinuria; HR: Heart Rate; RR: Respiratory Rate; BP: Blood Pressure; ER: Emergency Room; CT: Computed Tomography; G-CSF: Granulocyte-Colony Stimulating Factor

One of the rare infections linked to the use of eculizumab is *P. aeruginosa*. In 319 patients participating in post-marketing surveillance, Ninomiya et al. discovered that eculizumab therapy was linked to serious non-Neisserial infections such as pneumonia, sepsis, herpes zoster infection, and infection with an unknown pathogen. Complement deficient mice were more susceptible to *P. aeruginosa* infection than intact mice, according to Younger et al. The incident of *P. aeruginosa* infection was described by Kawakami et al. in their study as well [11]. The *P. aeruginosa* infection is described in Figure 2. 

Moraxella lacunata

**Figure 3:**
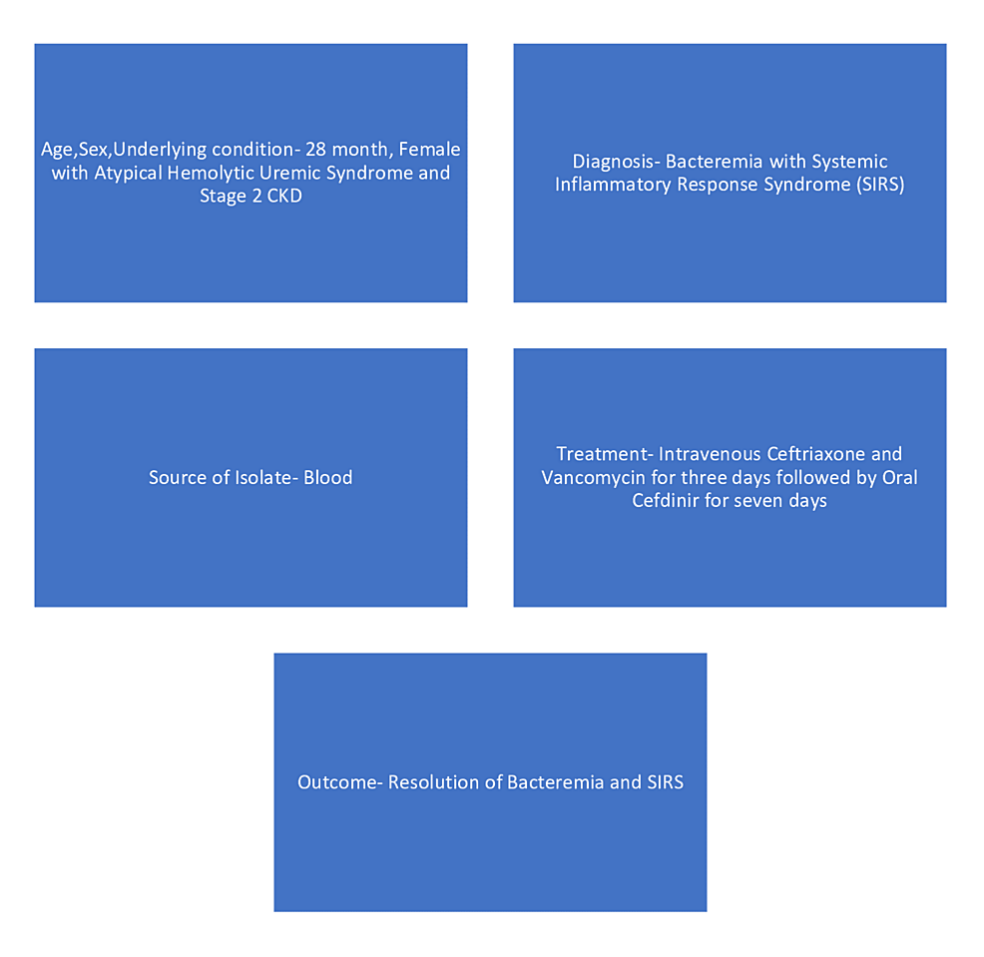
Description of Moraxella lacunata infection CKD: Chronic kidney disease; SIRS: Systemic inflammatory response syndrome

*Moraxella lacunata* is a commensal bacterium with a low pathogenicity potential that colonizes the upper respiratory system. It can, nevertheless, act as an opportunistic pathogen in an immunocompromised host. Bicoll et al.'s case report is the first to describe *Moraxella lacunata* bacteremia and SIRS in an immune-deficient child as a result of eculizumab treatment [10]. *Moraxella lacunata* infection is described in Figure 3.

Limitations

Our study did not include articles that were freely unavailable, in languages other than English, and written before the last 20 years. As a result, other probable infections are not mentioned.

## Conclusions

Our systematic review identifies a number of infections that have been linked to the use of eculizumab in individuals with PNH/aHUS. The bacteria that cause the infection are *N. meningitides*, *N. gonorrhea*, *N. flavescens (subflava)*, *N. cinerea*, *N. sicca*, *P. aeruginosa*, and *M. lacunata*. The therapeutic regimens for prophylaxis and treatment of infections are outlined in our article. These groundbreaking cases highlight the importance of a high susceptibility index among health care providers and patients for the various infections described above, resulting in early detection and consideration of empiric antibiotics, regardless of the patient's immunization status or prophylactic regimen. We propose more research in the form of a prospective cohort study in a large population of eculizumab-treated patients to seek for infections not included in our analysis.
